# Size‐Controllable Nickel Sulfide Nanoparticles Embedded in Carbon Nanofibers as High‐Rate Conversion Cathodes for Hybrid Mg‐Based Battery

**DOI:** 10.1002/advs.202106107

**Published:** 2022-03-03

**Authors:** Guilei Zhu, Guanglin Xia, Hongge Pan, Xuebin Yu

**Affiliations:** ^1^ Department of Materials Science Fudan University Shanghai 200433 China; ^2^ Xian Technol. Univ. Inst. Sci. & Technol. New Energy Xian 710021 China

**Keywords:** conversion mechanism, hybrid magnesium‐based battery, nickel sulfide, size‐controllable nanoparticles

## Abstract

The integration of highly‐safe Mg anode and fast Li^+^ kinetics endows hybrid Mg^2+^/Li^+^ batteries (MLIBs) a promising future, but the practical application is circumvented by the lack of appropriate cathodes that enable the realization of an enough participation of Mg^2+^ in the reactions, resulting in a high dependence on Li^+^. Herein, the authors develop a series of size‐controllable nickel sulfide nanoparticles embedded in carbon nanofibers (NiS@C) with synergistic effect of particle diameter and carbon content as the cathode material for MLIBs. The optimized particle size is designed to maximize the utilization of the active material and remit internal stress, and appropriate carbon encapsulation efficiently inhibiting the pulverization of particles and accelerates the ability of conducting ions and electrons. In consequence, the representative NiS@C delivers superior electrochemical performance with a highest discharge capacity of 435 mAh g^−1^ at 50 mA g^−1^. Such conversion cathode also exhibits excellent rate performance and remarkable cycle life. Significantly, the conversion mechanism of NiS in MLIBs is unambiguously demonstrated for the first time, affirming the corporate involvement of both Mg^2+^ and Li^+^ at the cathodic side. This work underlines a guide for developing conversion‐type materials with high rate capability and cyclic performance for energy storage applications.

## Graphical abstract

A series of size‐controllable nickel sulfide nanoparticles embedded in carbon nanofibers are successfully fabricated as the cathode material for MLIBs, which exhibit excellent rate performance and remarkable cycle life. Corresponding conversion mechanism has also been unambiguously demonstrated, affirming the corporate involvement of both Mg^2+^ and Li^+^ at the cathodic side and the irreversible transformation from NiS to Ni_2_S_3_.

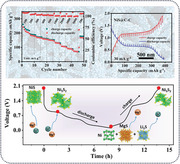

## 1 Introduction

The primary portable power source of multifarious applications is gradually occupied by lithium‐ion batteries (LIBs) since the invention in 1991, and the energy density at the cell level can implement 240 W h kg^−1^ on account of more than two decades of endeavor and innovation in materials and battery design.^[^ ^1^ ^]^ Unfortunately, intercalation anodes are extensively applied in current commercial LIBs due to the safety hazard caused by the dendrite formation of mestallic Li, where the ceiling of capacity and energy density is extremely untoward to break through when the theoretical boundary of intercalation chemistry is reached.^[^ ^2^ ^]^ In an attempt to tackle this issue, rechargeable metal batteries pairing metal anodes with diverse cathodes are endowed with broad prospects to further enhance energy density owing to the merits of higher capacity and lower reduction potential.^[^ ^3^ ^]^ Thanks to high theoretical volume specific capacity (3833 mA h cm^−3^ for magnesium versus 2046 mA h cm^−3^ for Li), low reduction potential (−2.4 V versus SHE), high earth abundance, high handling safety (high melting point; 651 °C for magnesium versus 180 °C for Li) and dendrite‐free deposition, the magnesium (Mg) anode stands out from other options.^[^ ^4^ ^]^ Nevertheless, Mg^2+^ with a bivalent nature renders a much more strong electrostatic interactions in solid state cathodes than monovalent Li^+^, which profoundly results in large voltage hysteresis, throwing a knotty problem for premature Mg‐ion batteries (MIBs).^[^ ^5^ ^]^ Therefore, the main effort should be put into the exploration of suitable cathode materials with satisfactory kinetics.

In terms of intercalation cathodes,^[^ ^6^ ^]^ the most extraordinary trait is that the crystal structure is maintained during the reaction process, which results in the advantage of small volumetric change (long cycle life) and the intrinsic drawback of limited ion intercalation/deintercalation (low theoretical specific capacity). The latter seriously curbs the further development and more attentions should be paid on scrutinizing conversion cathodes with the potential of high specific capacity. Typically, conversion‐type transition metal sulfides (CTMS), which excel at high theoretical capacity receive growing concern, but also have a big barrier in lifespan and rate performance generated by an inherently changing crystal structure. For unmodified CTMS, CuS microsphere has been verified as an effective cathode but presents an unsatisfactory specific capacity and cycle stability, accomplishing an initial capacity of 138 mAh g^−1^ at 20 mA g^−1^ and a remaining capacity of 40 mAh g^−1^ after 20 cycles.^[^ ^7^ ^]^ Consequently, the pivotal issue needs to be shifted to the rational design of CTMS to minimize the destruction caused by the large volumetric expansion, where previous studies mainly focus efforts on three directions. Interlayer‐expanded CTMS can well enlarge the dimension of ion diffusion channels and shield the coulomb interactions between Mg^2+^ and the host lattice anion, implementing a significantly enhanced capacity from 100 to 350 mAh g^−1^ at 200 mA g^−1^ via the intercalation of cetyltrimethylammonium bromide in CuS.^[^ ^8^ ^]^ A second general and effective approach to remit volumetric expansion is to construct reserved expansion space (nanoarray morphology,^[^ ^9^ ^]^ hollow or nanoporous feature,^[^ ^10^ ^]^ hierarchical architecture,^[^ ^11^ ^]^ et al.). For instance, He et al. demonstrate that flower‐like CoS with hierarchitectures and high crystallinity manifests good cycling stability, delivering 125.3 and 120.6 mAh g^−1^ in the 5th and 40th cycles at 50 mA g^−1^, respectively.^[^ ^9c^ ^]^ Additionally, the domain‐limiting strategy is also an eminent means to restrain the agglomeration of active materials and improve the electrical conductivity inside the cathode materials, including the introduction of elastic polymer or carbon encapsulation, or carriers with high specific surface area (such as graphene, MXene, CNTs, et al.), which are extensively investigated in LIBs and sodium ion batteries but few in MIBs. Although considerable progress has made in developing CMTS cathode materials for MIBs, to achieve high capacity, long cycle, and superior rate performance of such materials is still a big challenge.

To be more specific, high charge density intensifies electrostatic interactions between the host lattices, slowing Mg^2+^ diffusion and inhibiting reversible intercalation, which becomes an intractable hindrance for immature MIBs.^[^ ^12^ ^]^ Therefore, the development of MIBs is embarrassed by the sluggish kinetics of Mg^2+^ in active materials. In an attempt to tackle the above issue, Li^+^ with the merit of fast diffusion kinetics is introduced into MIBs via using a dual salt electrolyte, where Li^+^ is inserted into cathode and Li deposition/dissolution at the anode can also be well circumvented (due to the lower redox potential of Li than that of Mg).^[^ ^13^ ^]^ Commercial FeS_2_ shows almost negligible capacity in pure MIBs, but the reversible capacity can be greatly improved to 480 mAh g^−1^ at 0.1 C (89.4 mA g^−1^) when 1.5 m lithium borohydride was introduced, indicating a very effective method for boosting the electrochemical performance.^[^ ^14^ ^]^ Nevertheless, the occurrence of Li^+^ intercalation/deintercalation inevitably depends on the electrolyte amount, since it is employed as an ion reservoir to furnish Li^+^ and store Mg^2+^ during the reaction.^[^ ^15^ ^]^ More significantly, the confined solubility of Li salts in Mg^2+^/Li^+^ batteries (MLIBs) calls for exploiting new cathodes that can accommodate both Mg^2+^ and Li^+^ to reduce the above‐mentioned dependence. In recent years, tremendous progress has been made towards MLIBs on copper based chalcogenide cathodes, but for other CMTs, normally only Li^+^ can contribute capacity during charging and discharging process.^[^ ^14^, ^16^ ^]^

Representatively, nickel‐based sulfides with the benefits of excellent electrical, optical and magnetic properties, non‐toxicity, and being low cost have been extensively applied in various fields,^[^ ^17^ ^]^ especially in energy storage but few in MLIBs. Herein, we report a scalable strategy to synthesize a series of size‐controllable nickel sulfide nanoparticles embedded in carbon nanofibers, in which the particle diameter and carbon ratio were systematically regulated to maximumly optimize the synergistic effect. The optimal cathode with the superiority of high conductivity, great active‐material utilization and small internal stress achieves a highest discharge capacity of 435 mAh g^−1^ at a current density of 50 mA g^−1^, and exhibits outstanding rate capability and excellent cycling stability. More importantly, this work, with an excellent combination of ex‐situ and in‐situ XRD, unambiguously demonstrates the conversion mechanism of NiS in MLIBs for the first time, affirming the corporate involvement of both Mg^2+^ and Li^+^ at the cathodic side and the irreversible transformation from NiS to Ni_2_S_3_.

## 2 Results and Discussion

**Figure 1:**
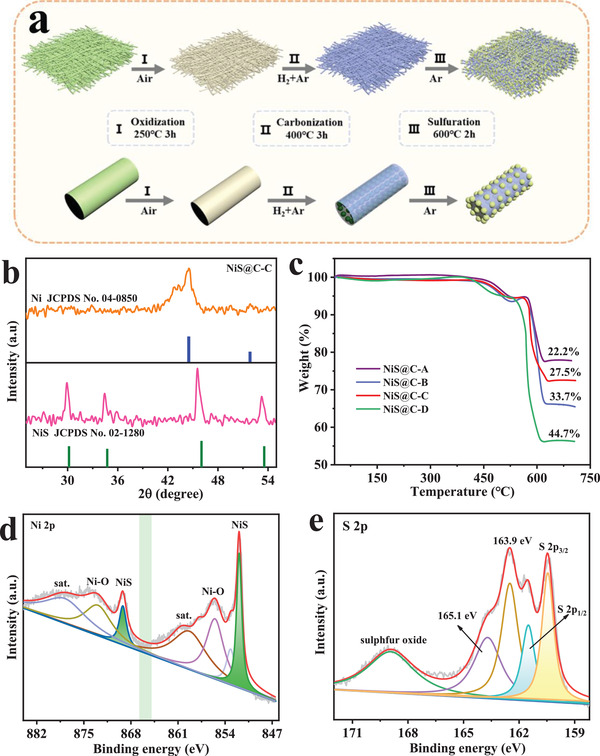
a) Schematic illustration of the fabrication for NiS@C. b) The XRD patterns of Ni@C precursor and NiS@C‐C. c) TGA curves of as‐prepared samples. XPS spectra for NiS@C‐C in the d) Ni 2p and e) S 2p.

The stepwise fabrication of NiS@C with a fibrous morphology is depicted in **Figure** **1**a, where the precursor consisted of Ni(NO_3_)_2_·6H_2_O and polyvinyl‐pyrrolidone in N,N‐dimethylformamide and ethanol (volume ratio; 1:1) solvent was obtained via a one‐step continuous electrospinning technique and the aforementioned material was synthesized through three subsequent steps. To be more specific, the fibrous precursor was first treated by oxidation in air to enhance the toughness of the material. Thereafter, a carbonization process and followed by sulfuration treatment were conducted in Ar atmosphere, in which the main target of the two‐step reaction is to remit the stress on the material during the reaction process, and can be prone to thorough vulcanization. It is worth mentioning that the as‐prepared NiS@C with an outstanding configuration of fine particles confined by conductive carbon is of great benefit to remit volumetric expansion and accelerate ionic conductivity. Moreover, counterparts with varying carbon content and NiS particle size were successfully fabricated via controlling the mass of original nickel source, carbonization temperature, and sulfuration temperature, more synthetic details are presented in Table S1, Supporting Information.

The X‐ray diffraction (XRD) patterns of as‐prepared carbide and sulfide samples are displayed in Figure 1b and Figure S1, Supporting Information. Obviously, two diffraction peaks at 44.5° and 51.8° corresponding to Ni phase (JCPDS No. 04–0850) all appear in the four Ni@C samples,^[^ ^18^ ^]^ where the signal of Ni@C‐A and Ni@C‐B exhibits similar intensity, which are far stronger than that for Ni@C‐C and Ni@C‐D. The reason for different intensities mentioned above can be ascribed to different carbonization temperature. After undergoing a sulfuration process, with the disappearance of the signal of Ni, new peaks at 30.2°, 34.7°, 46.0°, and 53.5° index to the hexagonal phase of NiS (*α*‐NiS; JCPDS No. 75–0613),^[^ ^19^ ^]^ which can be employed to describe NiS@C‐A, NiS@C‐B, and NiS@‐C materials, respectively. Intriguingly, NiS@C‐D exhibits few signals of NiS phase, which may be attributed to low crystallinity and small particle size caused by low sulfuration temperature. Furthermore, the phase of NiS@C‐D will be further analyzed in the following selected area electron diffraction (SAED). Since various synthetic conditions are prone to profoundly affect the carbon ratio and the carbon content is closely related to the conductivity and capacity, the thermogravimetric analysis (TGA) was conducted to accurately assess the carbon ratio. On the basis of the TGA curves in Figure 1c, the carbon contents in NiS@C‐A, NiS@C‐B, NiS@C‐C, and NiS@C‐D are 33.7%, 22.2%, 27.5%, and 44.7%, where the reason of higher carbon ratio for NiS@C‐A and NiS@C‐D is the high original PVP ratio. In addition, the lower carbonization temperature and sulfuration temperature results in less carbon in NiS@C‐A than in NiS@C‐D, and this phenomenon also can be extended to the difference between NiS@C‐B and NiS@C‐C.

The X‐ray photoelectron spectroscopy (XPS) measurement was conducted to further analyze the surface chemical composition and valence states of NiS@C‐C. As demonstrated in Figure S2a, Supporting Information, the survey spectrum testifies the existence of Ni, S, C, and O elements, where the signal of O is obviously owing to the surface oxidation in the air atmosphere. In terms of the high‐resolution Ni 2p and S 2p spectra, they are normalized by the difference between the measured C 1s (Figure S2b, Supporting Information; 283.4 eV) and the standard value (284.0 eV). The binding energies (BEs) at 853.3 and 870.6 eV (*Δ*_BE_ = 17.3 eV) can be ascribed to the Ni 2p_3/2_ and Ni 2p_1/2_ in NiS, respectively (Figure 1d).^[^ ^19^, ^20^ ^]^ Additionally, peaks at 854.5–859.3 and 874.3 eV with two corresponding satellite peaks (sat.) are allocated to the Ni 2p_3/2_ and Ni 2p_1/2_ in oxidized species (such as Ni_3_O_2_, etc.).^[^ ^21^ ^]^ The S 2p spectrum in Figure 1e shows two pairs of peaks and one oxidized peak (170.3 eV; sulfur oxide), in which the BEs located at 161.8 and 163.0 eV (*Δ*_BE_ = 1.2 eV) corresponding to S 2p_3/2_ and S 2p_1/2_ are the signal of S^2−^ in NiS and another pair of peaks at 163.9 and 165.1 eV can be identified to the C—S bond in the composite.^[^ ^22^ ^]^ The above analysis indicates that the primary active material is NiS phase, which is consistent with the XRD results.

**Figure 2:**
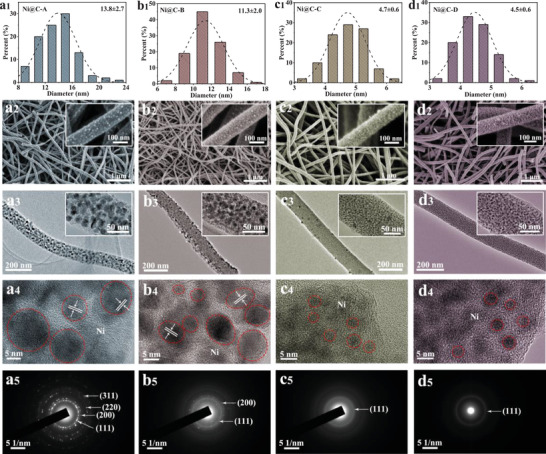
a1–d1) Corresponding histogram of the Ni size distributions for Ni@C‐A, Ni@C‐B, Ni@C‐C, and Ni@C‐D. a2–d2) SEM images, a3–d3) TEM images, a4–d4) HRTEM images, and a5–d5) SAED patterns of Ni@C‐A, Ni@C‐B, Ni@C‐C, and Ni@C‐D.

The scanning electron microscopy (SEM) images of raw fibers and oxidized fibers are presented in Figure S3, Supporting Information, elucidating a smooth surface and an unaltered fibrous morphology after oxidization process in Air atmosphere. **Figure** **2** clearly depicts the morphological and structural feature of as‐prepared carbide samples. The representative average diameters of Ni@C‐A, Ni@C‐B, Ni@C‐C, and Ni@C‐D are calculated to 13.8, 11.3, 4.7, and 4.5 nm (Figure 2a1–d1), where the carbonization temperature of Ni@C‐A and Ni@C‐B (500 °C) is higher than that of Ni@C‐C and Ni@C‐D (400 °C), resulting in larger particle diameters. The SEM images of corresponding four materials in Figure 2a2–d2 manifest the well maintained fibrous structure and fibrous size, but show a visibly rough surface. More significantly, the diameter of different samples is analogous (≈130 nm), revealing that carbonization temperature has little influence on fiber size. For the sake of further studying the Ni particles in carbon fiber, the transmission electron microscopy (TEM) was employed to investigate Ni@C (Figure 2a3–d3). Obviously, the particle size of Ni@C‐A is slightly larger than that of Ni@C‐B, but is much larger than that of Ni@C‐C and Ni@C‐D. Admirably, the ultrafine Ni particles are evenly dispersed in carbon fibers, which is beneficial to subsequently complete sulfuration. The corresponding high‐resolution TEM (HRTEM) images in Figure 2a4–d4 uncover the crystallization characteristics of Ni particles that is further verified by the SAED (Figure 2a5–d5).

**Figure 3:**
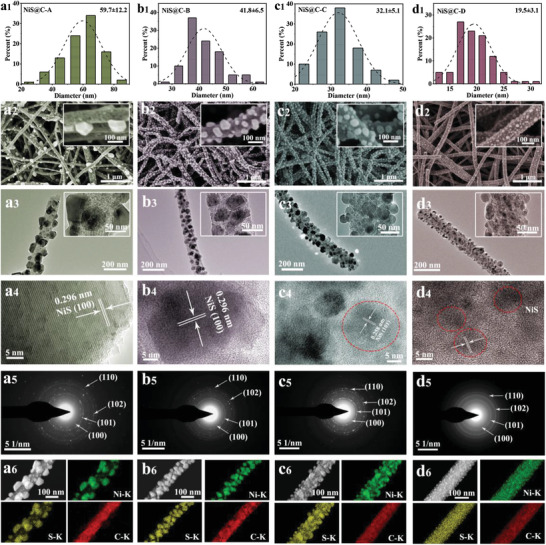
a1–d1) Corresponding histogram of the NiS size distributions, a2–d2) SEM images, a3–d3) TEM images, a4–d4) HRTEM images, a5–d5) SAED patterns, and a6–d6) STEM images and the corresponding elemental mappings of Ni, S, and C for NiS@C‐A, NiS@C‐B, NiS@C‐C, and NiS@C‐D.

The final products are fabricated via a simple sulfuration process of the Ni@C precursors under Ar atmosphere. The typical histograms of the NiS size distributions are clearly demonstrated in **Figure** **3**a1–d1, manifesting the average sizes of 59.7, 41.8, 32.1, and 19.5 nm for NiS@C‐A, NiS@C‐B, NiS@C‐C, and NiS@C‐D, respectively. It is worth mentioning that the standard deviation of NiS@C‐A is far larger than that of other three samples, testifying an uneven particle size. On the contrary, the NiS particles in NiS@C‐B, NiS@C‐C, and NiS@C‐D have relatively uniform particle size distribution. The results showed that homogenous NiS particles could be obtained more easily with a sulfuration time of 2 h rather than 3 h. From the SEM images of as‐prepared four sulfides displayed in Figure 3a2–d2, the conclusion that carbon fiber morphology is perfectly maintained can be drawn. Whereas, the distribution of NiS particles is markedly different, in which the distribution and the size of NiS particles in NiS@C‐A are obviously irregular compared to the other three products. In addition, TEM images were collected to further analyze the NiS particles in carbon fibers, and the result is well in accordance with the SEM analysis. Combining the experimental synthesis conditions and the morphology results, the main reason for the large particle size and uneven particle distribution in NiS@C‐A is the long sulfuration time. Moreover, the particle size of precursors and the sulfuration temperature are also the pivotal factors to greatly affect the NiS particle size. The crystallinity of individual NiS particles is probed by HRTEM measurement (Figure 3a4–d4), where the clear lattice fringe of 0.296 nm for NiS@C‐A and NiS@C‐B is well indexed to the (100) plane of NiS phase and the crystallization feature of NiS@C‐C and NiS@C‐D is also evident. Besides, the SAED images in Figure 3a5–d5 present an array of diffraction rings, which can be assigned to the (100), (101), (102), and (110) crystal planes of NiS phase. Attentively, the structure of NiS@C‐D is precisely affirmed here. Figure 3a6–d6 depict the STEM images and the corresponding elemental mappings of Ni, S, and C for four samples, furnishing more details of the elemental distribution in NiS@C‐A, NiS@C‐B, NiS@C‐C, and NiS@C‐D materials.

**Figure 4:**
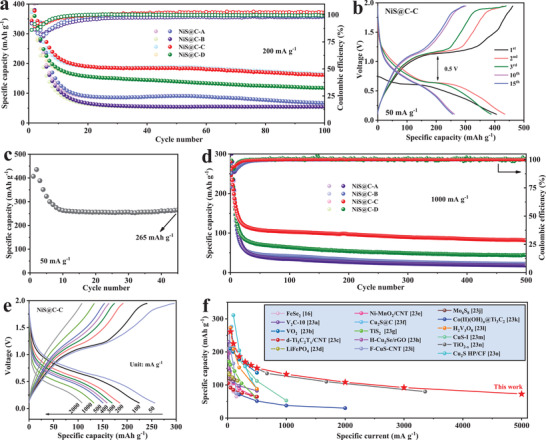
a) Cycling performance and CE of NiS@C‐A, NiS@C‐B, NiS@C‐C, and NiS@C‐D at a current density of 200 mA g^−1^. b) Galvanostatic charge–discharge voltage profiles of NiS@C‐C at 50 mA g^−1^. c) Cycling performance of NiS@C‐C at a current density of 50 mA g^−1^. d) Cycling performance and CEs of NiS@C‐C at 1000 mA g^−1^. e) Galvanostatic charge–discharge voltage profiles of NiS@C‐C at various current density. f) Comparison of rate capability of the state‐of‐the‐art cathodes for reported Mg^2+^/Li^+^ hybrid battery systems.

To further investigate the influence of carbon content and NiS particle size on the Mg^2+^/Li^+^ storage performance, the electrochemical properties of NiS@C‐A, NiS@C‐B, NiS@C‐C, and NiS@C‐D are inquired (**Figure** **4**). Figure S4, Supporting Information, describes the galvanostatic charge–discharge voltage profiles of NiS@C‐A, NiS@C‐B, NiS@C‐C, and NiS@C‐D at 200 mA g^1^, expressing consistent discharge voltage plateau and different capacity decay trend. Specifically, NiS@C‐B shows obviously higher initial discharge capacity (521 mAh g^−1^) than that of NiS@C‐A, NiS@C‐C, and NiS@C‐D, which can be ascribed to highest NiS ratio in NiS@C‐B (77.8%). Unfortunately, the capacity rapidly declined in the following cycles, and the discharge capacity decreases to 90 mAh g^−1^ at the 20th cycle with 98% coulombic efficiency (CE). Furthermore, the discharge capacity of NiS@C‐A has a lower value (66 mAh g^−1^) than that of NiS@C‐B after undergoing 20th cycles. In sharp contrast, the reversible capacity of NiS@C‐C and NiS@C‐D is much larger than that of NiS@C‐A and NiS@C‐B, where NiS@C‐C reveals a most eminent performance, including capacity (200 mAh g^−1^) and CE (99.9%). The above capacity comparison is also clearly demonstrated in Figure 4a. For the comparison of NiS@C‐A, NiS@C‐B, and NiS@C‐C, the reversible capacity enhances with the decrease of NiS particle size (from 60 to 32 nm). Such a case can be made clear by the small particle diameter that is more liable to magnify contact surface between electrolyte and active materials, alleviate internal strain and shorten electron transport distance. Attentively, the diameter of NiS@C‐D is smaller than that of NiS@C‐C, but implements a lower reversible capacity, attributing to a much lower NiS ratio than that of NiS@C‐C (72.5% for NiS@C‐C; 55.3% for NiS@C‐D). It is widely accepted that carbon coating can effectively promote the electron‐conducting ability in internal materials and confine the aggregation between particles, but large amounts of carbon can also reduce the proportion of active materials and thus greatly lower the theoretical capacity. Additionally, the typical cyclic voltammetry (CV) curves of four sulfides electrodes at a scan rate of 0.1 mV s^−1^ in Figure S5, Supporting Information, further verify the above results. In consequence, NiS@C‐C is the optimized electrode, and further research will be carried out with this focus in the following work.

Figure 4b displays the galvanostatic charge–discharge voltage profiles of NiS@C‐C at 50 mA g^−1^, achieving a high initial discharge capacity of 407 mAh g^−1^ and a low electrochemical polarization. More significantly, the shape of the charge–discharge curves is well maintained after undergoing 10 cycles, which uncovers a good reversibility. It is also worth noting that the discharge capacity of NiS@C‐C tends to be stable in the subsequent cycles and implements a superior capacity of 265 mAh g^−1^ after experiencing 45 cycles, suggesting an excellent cycle stability (Figure 4c). Thanks to the change of the crystal structure of the host material during repeated charging and discharging cycles, the conversion cathode material will experience a much larger volumetric expansion than that of intercalation materials, which results in that the rate performance and long cycle life become an intractable hindrance to overcome. In this work, the NiS nanoparticles are well anchored in carbon fibers, which is great beneficial for alleviating the pulverization of active materials and facilitating ion diffusion. Therefore, NiS@C‐C exhibits an excellent cycle stability and high CEs (99.9%), maintaining the electrochemical activity after repeating 500 cycles at 1000 mA g^−1^. Besides, an analogous tendency can be seen at the NiS@C‐A, NiS@C‐B, and NiS@C‐D electrodes, but demonstrate obviously low specific capacity. On the other hand, the rate capability of four electrodes was recorded at different current densities from 0.05 to 5 A g^−1^. As a result, NiS@C‐C delivers an outstanding rate capability (Figure S6a, Supporting Information), with the discharge capacities of 261, 225, 185, 170, 160, 151, 133, 108, 92, and 73 mAh g^−1^ at 50, 100, 200, 300, 400, 500, 1000, 2000, 3000, and 5000 mA g^−1^, respectively. Meanwhile, the discharge–charge voltage profiles at various current densities in Figure 4e show the invariable shape of the curves, but display increased polarization voltage and decreased discharge capacities. The primary reason can be summed up to that the electrochemical reaction rate is faster than the diffusion rate of Mg^2+^/Li^+^ and is slower than the migration rate of electrons. As expected, the rate capabilities of NiS@C‐A and NiS@C‐B are far poor than that of NiS@C‐C, attaining low discharge capacities of 23 and 27 mAh g^−1^ at a current density of 5000 mA g^−1^, respectively. Such inferior performance can be generated by the much sluggish diffusion rate of Mg^2+^/Li^+^ inside the electrode materials with a large NiS particle diameter. Compared with the reported literatures, the performances achieved above are not very outstanding in terms of specific capacity, but such strategy can be utilized as a reference in enhancing the rate performance, especially for the conversion materials. As shown in Figure 4f, the rate capability is obviously more superior than most of investigated conversion cathodes and lots of intercalation cathodes in hybrid MLIBs.^[^ ^16^, ^23^ ^]^ Furthermore, in consequence of high carbon content and the capacity calculated by total mass, the performance of bare carbon nanofiber in MLIBs was also tested to probe the capacity contribution of carbon coating. As depicted in Figure S7, Supporting Information, the bare carbon nanofiber delivers a reversible capacity of 37 mAh g^−1^ at 200 mA g^−1^, and the corrected capacities of NiS@C‐C and NiS@C‐D display a much smaller gap than that of total capacities, in which the difference may be ascribed to the synergistic effect between crystallinity, particle size, and carbon content. The reversible specific capacity based on the mass of NiS was calculated to be 353 mAh g^−1^ at 50 mA g^−1^ according to the method mentioned above.

The electrochemical impedance spectra (EIS) measurement in Figure S7, Supporting Information, was performed on fresh NiS@C‐A, NiS@C‐B, NiS@C‐C, and NiS@C‐D electrodes to further investigate the intrinsic factors of performance differences. The EIS plots of pristine cathodes show semicircles in high frequency and sloping lines in low frequency, which corresponds to charge transfer resistance (*R*_ct_) at the electrode/electrolyte interface and the Mg^2+^/Li^+^ diffusion resistance within the electrode materials, respectively.^[^ ^24^ ^]^ The NiS@C‐C electrode presents much lower resistance values than that of other three cathodes, testifying that optimized configuration of carbon and particle diameter is in favor of generating conductive fiber for accelerating electron and ions diffusion transfer, thus beneficial to the capacity and rate capability. Besides, the galvanostatic intermittent titration technique (GITT) was employed to offer further insight into the variation in Mg^2+^/Li^+^ solid‐state diffusion during charge and discharge process. In the GITT curve, NiS@C‐C delivers a capacity of 393 mAh g^−1^ (Figure S8a, Supporting Information). Thereafter, the Fick's second law was utilized to calculate the diffusion coefficient (*D* ^GITT^).^[^ ^25^ ^]^ As‐gained diffusion coefficients at different voltages are shown in Figure S8b, Supporting Information, in which the values exhibit a relatively slow ions migration rate at plateau stage. However, the changing trend in the charge process is in accord with that in the discharge process, and the values in each stage are approximate, indicating that the *D* ^GITT^ of the conversion cathode in the system is slightly related to the ions concentration, but deeply influenced by the components.

The CV measurement at various sweep rates from 0.1 to 1.0 mV s^−1^ was performed to systematically investigate electrode reaction kinetics of optimized NiS@C‐C (Figure S9, Supporting Information). Theoretically, the voltammetric response of a cathode material can be evaluated as following formulas:

(1) $$I\left(V\right)=av^{b}$$

(2) $$\log i\left(V\right)=b\log v+\log a$$

where the measured current (*i*) and the scan rate (*v*) obey a power law relationship, and *a* and *b* are adjustable parameters.^[^ ^25^, ^26^ ^]^ The peak current *i* varies as *v* ^1/2^ or *v* (that is, *b* = 0.5 or 1), elucidating that the electrochemical system predominates the diffusion process or capacitive‐controlled process, respectively. For one pair of cathodic and anodic response in Figure S9a, Supporting Information, the relationship between log(*i*) and log(*v*) is presented in Figure S9b, Supporting Information. The *b* values determined by the slopes of the two redox peaks were calculated to be 0.63 and 0.90, suggesting that the reaction is controlled by both diffusive and capacitive processes. To be more specific, the current contributions from the capacitance effect (*k_1_v*) and diffusion process (*k_2_v^1/2^*) can be quantified at a fixed scan rate via the following equation:

(3) $$i\left(V\right)=k_{1}v+k_{2}v^{1/2}$$

As a result, the pseudocapacitive contribution of stored charges is calculated as 75.5% at 1 mV s^−1^ (Figure S9c, Supporting Information). Furthermore, as displayed in Figure S9d, Supporting Information, the contribution ratios of capacitive process at 0.2, 0.4, 0.6, and 0.8 mV s^−1^ are 58.7%, 64.1%, 68.2%, and 72.0%, respectively. The results indicate that the capacitive contribution in the total capacity is close to the diffusive contribution at a small sweep rate of 0.2 mV s^−1^, while the capacitive contribution ratio rapidly enhances with the increase of scan rate, which is the reason why NiS@C‐C electrode has an outstanding rate performance in MLIBs.

Additionally, the NiS@C‐C electrode was also performed in the pure APC electrolyte to further study the role of Li^+^ in the hybrid system. As shown in Figure S11, Supporting Information, the NiS@C‐C cathode delivers a low capacity at 50 mA g^−1^, indicating poor electrochemical performance in pure MIBs. A significant impact of Li^+^ is to effectively reduce the charge transfer impedance and facilitate the ion diffusion within electrode, which is adequately demonstrated via the EIS plots of NiS@C‐C in MIBs and MLIBs (Figure S12, Supporting Information). More specifically, the GITT was also conducted to provide more evidences (Figure S13, Supporting Information). Given that the NiS@C‐C cathode shows low discharge capacity, such measurement was tested at a smaller current pulse of 10 mA g^−1^ for a shorter time (10 min) followed by an open‐circuit relaxation of 30 min. Figure S13c, Supporting Information, reveals that the diffusion coefficients in MIBs are much lower than that in MLIBs. In consequence, it can be concluded that the addition of Li^+^ can effectively reduce the impedance and enhance the diffusion rate. As for the reaction between Li^+^ and active materials, it will be discussed later. Thereafter, the electrochemical property of NiS@C‐C in LIBs was also been investigated. Figure S14, Supporting Information, displays that NiS@C‐C accomplishes a high initial discharge capacity of 572 mAh g^−1^ at 200 mA g^−1^, but also experiences an obvious decline during the early cycles. Amusingly, the NiS@C‐C electrode also exhibits a good cycling stability in LIBs (219 mAh g^−1^ after 65 cycles), manifesting that NiS@C‐C also has good Li storage property.

**Figure 5:**
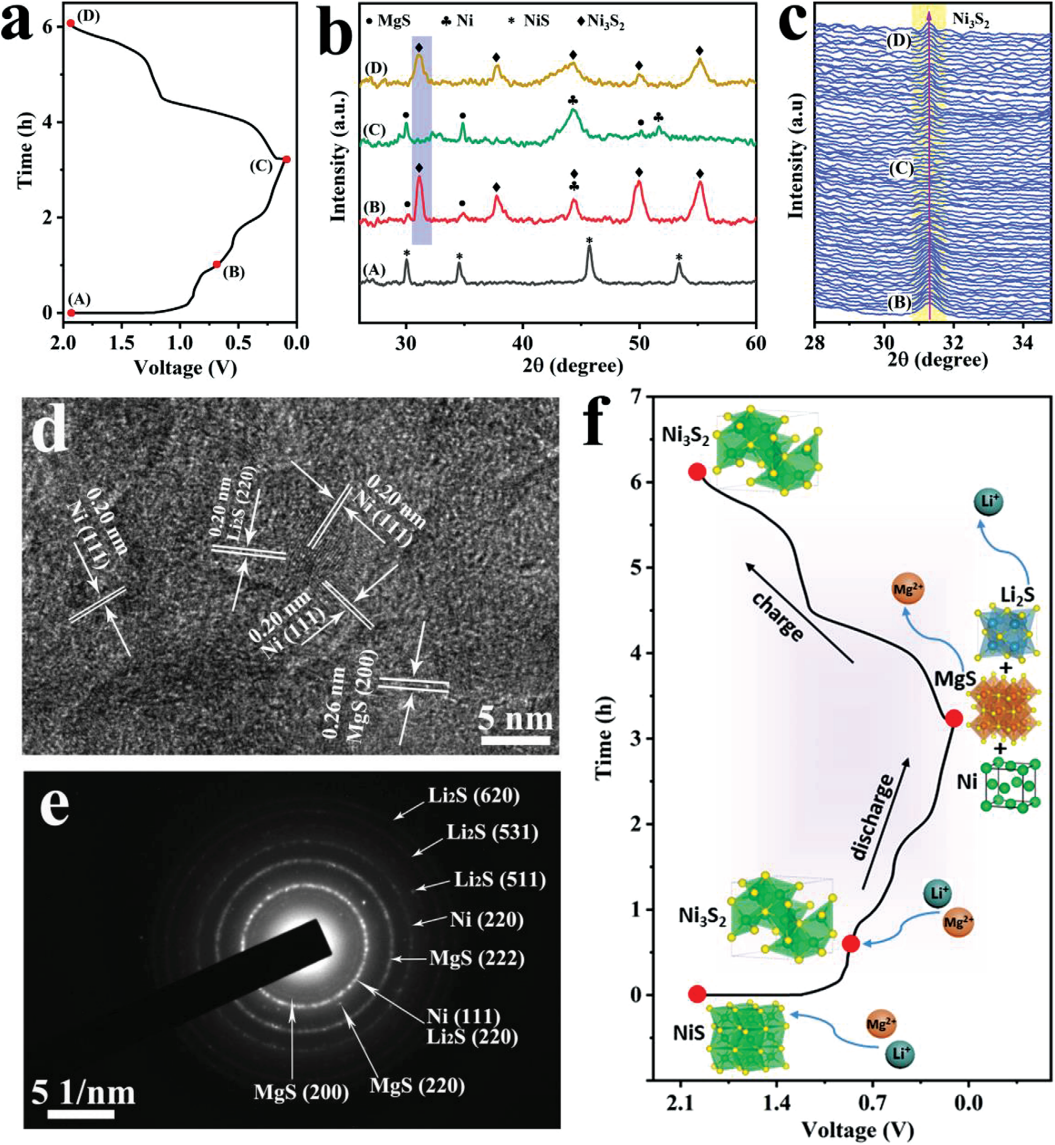
a) Galvanostatic charge and discharge curve of the NiS@C NPs/CC cathode at 200 mA g^−1^. b) Ex situ XRD patterns of the NiS@C NPs/CC electrode at different electrochemical states: A) as‐prepared, B) first discharge to 0.7 V, C) first discharge to 0.1 V, D), first charge to 1.9 V. c) In‐situ XRD patterns of the highlighted part in (b). d) HRTEM image and e) SAED pattern of NiS@C NPs at the fully discharged state. f) A schematic of the reaction mechanism for NiS.

For the development of a battery, the reasonable design of cathode materials, the calculation of theoretical capacity value and the assembly of full cell are inseparable from the storage mechanism, hence storage behavior is an indispensable investigation for developing a cathode material. **Figure** **5**a provides the initial charge–discharge curves of NiS@C/CC and corresponding sites to be probed by ex situ XRD. The ex situ XRD results of NiS@C/CC in MLIBs at the states marked in Figure 5a are presented in Figure 5b. First of all, the pristine powder material corresponding to (A) site in Figure 5a well matches with NiS phase with a space group of P63/mmc, which is perfectly in accordance with NiS@C‐C. When discharged to (B) site (0.7 V), the remarkable thing is that all original characteristic peaks are completely substituted by new peaks located at 31.1°, 37.8°, 44.3°, 50.1°, and 55.2°, which indexes well to (110), (003), (202), (211), and (211) crystal face of Ni_3_S_2_ phase (JCPDS no. 44–1418) with a space group of R32 (155).^[^ ^27^ ^]^ More significantly, weak peaks at 29.7° and 34.5° can be identified as the characteristic peaks of MgS (JCPDS no. 35–0730) with a space group of Fm‐3m (225).^[^ ^28^ ^]^ Above results verify that the complete transformation of NiS into Ni_3_S_2_ (from site (A) to site (B)) accompanied by the generation of MgS, which affirms that Mg^2+^ is involved in the reaction at the initial discharge process. After a deeper discharge to 0.1 V (site (C)), with the vanishment of the peaks for NiS phase, the signal of MgS is obviously strengthened and two peaks at 44.5° and 51.8° ascribed to metal Ni (JCPDS no. 04–0850) appear, where the peak at 44.5° has an overlap with Ni_3_S_2_ and CC substrate. The conclusion that Ni_3_S_2_ is converted into Ni and MgS can still be drawn even though a peak overlaps. Last but not least, with the disappearance of the peaks for MgS and Ni, the peaks of Ni_3_S_2_, rather than NiS, reappear obviously, revealing an irreversible conversion from NiS to Ni_3_S_2_. To gain more convincing evidence, in situ XRD was employed to collect the signal of the stage from site (B) to site (D) (highlighted in Figure 5b) that is the strongest peak. The results in Figure 5c make the summary obtained by the above ex situ XRD more credible. As the electrolyte contains Li salt, it is obligatory to explore whether Li^+^ participate in the cathodic reaction. The HRTEM and SAED of fully discharged NiS@C depicted in Figures 5d and 5e adequately testify the generation of Li_2_S. Furthermore, the high‐resolution XPS spectra of Li 1s and Mg 2p were employed to verify the involvement of Li^+^. Figure S15, Supporting Information, makes clear that both Mg^2+^ and Li^+^ present obvious signals when discharged to 0.1 V, which further confirms the participation of Li^+^ in the cathodic reaction during the discharge and charge process. Above XRD, HRTEM, SAED and XPS analyses illustrate that a two‐step conversion reaction can be summarized to account for the Mg^2+^ and Li^+^ storage mechanism of NiS cathode (Equations (4) and (5), in which the former is an irreversible process):

(4) $$\begin{array}{ccc} & & 3\left(x+y\right)\mathrm{NiS}+xMg^{2+}+y2Li^{+}+2\left(x+y\right)e^{-}\\ & & \;\to \left(x+y\right)Ni_{3}S_{2}+x\mathrm{MgS}+yLi_{2}S \end{array}$$

(5) $$\begin{array}{ccc} & & 0.5\left(x+y\right)Ni_{3}S_{2}+xMg^{2+}+2yLi^{+}+2\left(x+y\right)e^{-}\\ & & \;↔1.5\left(x+y\right)\mathrm{Ni}+x\mathrm{MgS}+yLi_{2}S \end{array}$$

From the above analysis results, the working mechanism of hybrid MLIBs in this work is illustrated in Figure S10, Supporting Information, where the deposition/dissolution of Mg takes place on the anode side and the insertion/extraction of both Mg^2+^ and Li^+^ dominates cathodic reaction. In addition, the transformations involving phase and crystal structure during the charge and discharge process of NiS cathode for MLIBs are graphically demonstrated in Figure 5f, indicating that NiS has a good Mg storage property.

Attentively, the irreversible process revealed by the conversion mechanism may admirably be employed to elucidate the capacity degradation during the initial cycles. Conceivably, the above‐mentioned irreversible reaction involves stoichiometric transformations, which have the tendency to result in the formation of soluble polysulfides. For the sake of testifying the above conjecture, the inductively coupled plasma mass spectrometry (ICP‐MS) analysis was performed to measure the sulfur content in the separator after various cycle numbers to confirm the polysulfide dissolved in the electrolyte. The result in Figure S17, Supporting Information, indicates that the polysulfide mainly occurs in the initial cycles, uncovering the primary reason for capacity fading. Moreover, the ultraviolet–visible (UV–vis) spectra in Figure S18, Supporting Information, furnish more evidence to further study the types of polysulfides produced by the conversion of NiS, in which the categories of polysulfides keep unchanged with the increase of cycle number. Nevertheless, by combining with ICP‐MS result, a conclusion can be drawn that polysulfides are mainly on the cathodic surface after having a rest of 24 h, while more polysulfides are adsorbed to the separator after withstanding 10 cycles. On the other hand, the presence of polysulfide may also lead to shuttle effects, which can be validated by the energy‐dispersive X‐ray spectroscopy (EDX) analysis and corresponding elemental mappings. Figure S19, Supporting Information, implies little sulfur content (0.05%, within the margin of error), manifesting that few polysulfides shuttle to the anode side with ions during the discharge and charge processes. Consequently, the irreversible reaction mainly generates the capacity decay during the early cycle.

**Figure 6:**
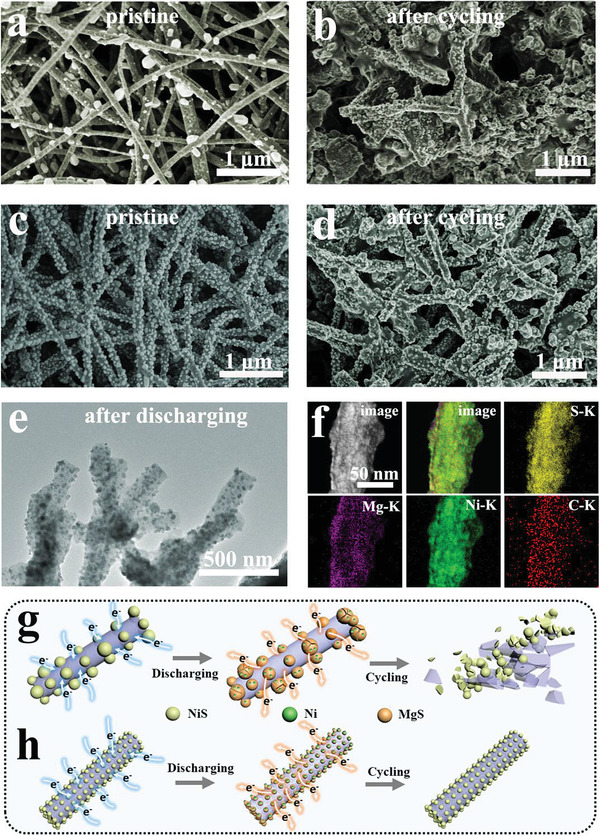
The SEM images of NiS@C‐A a) before and b) after cycling test. The SEM images of NiS@C‐C c) before and d) after cycling test. e) The TEM image of NiS@C‐C after discharging test. f) The STEM image and element maps of NiS@C‐C after cycling test (Ni, S, Mg, and C). Schematic of the structural stability of g) NiS@C‐A and h) NiS@C‐C during the repeated cycles.

**Figure** **6**a–d depicts the morphologies of NiS@C‐A and NiS@C‐C before and after 50 cycles at 200 mA g^−1^, furnishing sufficient evidence for investigating the structural stability. To be more specific, the NiS particles in NiS@C‐A were detached from the carbon fibers and abundant carbon fibers were fractured after undergoing a cycling process, while the NiS particles and carbon fibers in NiS@C‐C were perfect kept. Moreover, the TEM image and element mappings of Ni, S, Mg, and C for NiS@C‐C are also demonstrated in Figure 6e,f, manifesting the integrity of the internal structure and the uniform distribution of each element in the fibers. Additionally, the morphology of NiS@C‐D after cycling test in Figure S11, Supporting Information, also testifies the analogous advantages of small particles and uniform distribution. For the sake of deeply understand the relationship between capacity retention and particle distribution, the schematic diagrams of discharging and cycling processes for NiS@C‐A and NiS@C‐C are clearly demonstrated in Figures 6g and 6h. It is worth noting that the particles with large size are more prone to crack because of the larger internal stress and volume expansion during reaction process. On the other hand, the fibers embedded with uneven particles are more likely to fracture due to the stress concentration caused by a non‐uniform force. As a consequence, the above two factors may greatly reduce the utilization rate of active substances, resulting in a rapid capacity decay. In sharp contrast, cathode materials with the traits of small diameter and uniform distribution can better maintain the integrity of the structure, leading to higher material utilization, thus achieving higher reversible capacity and longer lifespan. According to the above discussion, the eminent cycle performance and rate capability of the NiS@C‐C can be mainly ascribed to the uniformity and carbon encapsulation.

## 3 Conclusions

In summary, a scalable strategy was designed to synthesize a series of size‐controllable nickel sulfide nanoparticles embedded in carbon nanofibers, which were employed as cathode material for systematically investigating influence of NiS particle diameter and carbon content on the hybrid Mg^2+^/Li^+^ storage. Experimental results manifest that the cathode material with a perfect configuration of small and uniform NiS particle size and appropriate carbon content avails to maximize the utilization of active material and relieve the stress inside the cathode electrode. Most significantly, the legible conversion mechanism of NiS in MLIBs is explicitly demonstrated by an unexceptionable combination of ex‐situ and in‐situ XRD, verifying the corporate involvement of both Mg^2+^ and Li^+^ at the cathodic side and the irreversible transformation from NiS to Ni_2_S_3_. Overall, we anticipate this work would highlight the potential of novel conversion cathode materials designed with high theoretical capacity and low cost features to boost the lifespan and rate capability in magnesium‐based batteries.

## 4 Experimental Section

### Synthesis of the Electrolyte

All‐phenyl complex (APC)‐based hybrid electrolyte was prepared according to reported method.^[^ ^23o^ ^]^

### Synthesis of NiS@C‐C

NiS@C‐C was synthesized through an electrospinning technique followed by stepwise thermal annealing processes. In a typical fabrication, polyvinylpyrrolidone (PVP, 0.17 g, Mw ≈1 300 000) and nickel nitrate hexahydrate (Ni(NO_3_)_2_ 6H_2_O, 0.101 g) were dispersed in 2.5 mL mixed solvent of ethanol and *N*,*N*‐dimethylformamide (DMF) (volume ratio; 1:1) with vigorous stirring for 24 h to obtain a homogeneous spinning dope, which was used as the precursor for electrospinning. Thereafter, the homogenous solution was poured into a 10 mL plastic syringe equipped with an 18‐gauge blunt‐tipped needle, which was controlled to be a slow low rate of 250 µL h^−1^ by a syringe pump (Longer, TJP‐3A, China). Besides, copper foil was horizontally placed and grounded to collect the as‐electrospun nanofibers, and kept an optimal distance between the needle and the collector of ≈15 cm. A high voltage of 12 kV was provided by a high‐voltage power supply to initiate the electrospinning.

Subsequently, the as‐collected fibers require to be quickly stored in an anhydrous environment, or timely oxidized in air at 250 °C with a heating rate of 2 °C min^−1^ for 3 h. Then, Ni@C‐C nanofiber material was prepared via a simple reduction process at 500 °C with a heating rate of 2 °C min^−1^ for 3 h in H_2_/Ar atmosphere. Finally, the resulting Ni@C‐C and appropriate sulfur powder were put into two separate porcelain boats. After flushed with Ar, the temperature was elevated to 600 °C at a rate of 2 °C min^−1^ and then kept for 2 h. The NiS@C‐C cathode will successfully acquire after naturally cooling to ambient temperature. Bare carbon nanofiber, NiS@C‐A, NiS@C‐B, and NiS@C‐D were synthesized via varying the Ni resource mass, carbonization temperature, sulfuration temperature, or sulfuration time, as illustrated in Table S1, Supporting Information.

### In Situ Generation of Binder‐Free NiS/CC Cathode

For mechanism analysis, we also prepared binder‐free NiS/CC cathode. 0.2 m Ni(NO_3_)_2_ solution (24 mL) and 0.267 m C_6_H_5_COONa solution (36 mL) were adequately mixed at room temperature. Subsequently, the green solution and a piece of CC (2 cm × 4 cm) were transferred to a 50 mL Teflon‐lined stainless steel autoclave. The autoclave was sealed and maintained at 95 °C for 4 h. After the autoclave cooled down at room temperature, the resulting precursor was taken out and washed with distilled water and ethanol, followed by drying at 80 °C. Whereafter, the resulting precursor and appropriate sulfur powder were put into two separate porcelain boats. After flushed with Ar, the temperature was elevated to 500 °C at a rate of 2 °C min^−1^ and then maintained for 2 h, and then naturally cooled to ambient temperature. The mass loading for NiS@C NPs/CC was ≈4.0 mg cm^−2^.

### Materials Characterizations

The phase structures were measured by XRD (D8 Advance, Bruker AXS Corporation) with Cu K*α* radiation (*λ* = 1.5418 Å). XPS was performed on a Thermo Scientific K‐Alpha^+^ spectrometer equipped with a single Al K*α* X‐ray source (1486.6 eV) operating at 100 W. Samples were studied under vacuum (*P* < 10^−8^ mbar) with a pass energy of 150 eV (survey scans) or 25 eV (high‐resolution scans). All binding energies were calibrated by using the contaminant carbon (C 1s = 284.0 eV) as a reference. The morphologies and microstructures of the original fibers, oxidized fibers, carbonized fibers, and sulfurized fibers and the differences between fresh materials and cycled electrodes were investigated using a field emission scanning electron microscope (FE‐SEM; JEOL7500FA, Tokyo, Japan). Energy‐filtered TEM (JEOL 2011 F, Tokyo, Japan) was further used to investigate the nanostructure, the crystallinity, and the elemental distribution. Thermogravimetric analysis (TGA, Netzsch STA 449 F3) connected to a mass spectrometer (MS, Hiden HPR 20) was employed to quantize the carbon ratios in various materials, where the ramp rate was kept at 10 °C min^−1^ during all the measurement. ICP‐MS analysis was conducted on Thermo Scientific iCAP6300. The ultraviolet–visible (UV–vis) absorption spectroscopy (Lamda 35, Perkin‐Elmer, USA) was performed for the solution after soaking the cycled separator and sulfide electrodes.

### Electrochemical Measurements

The electrochemical performances were tested with the use of 2032 coin‐type cells in an argon‐filled glove box. The electrodes were fabricated by coating slurries containing NiS@C active materials (80% wt%), super‐P acetylene carbon (10% wt%), and polyvinylidene fluoride (PVDF; 10% wt%) on stainless steel foil. The areal mass loading of active material is ≈1 mg cm^−2^. Additionally, as‐obtained binder‐free NiS/CC cathode material was punched into a 12 mm disk as working electrodes. Magnesium metal and glass fiber were used as counter electrode and separator, respectively. APC‐LiCl, which has the merits of low price and high coulombic efficiency, was employed as electrolyte. For MIBs, pure APC/THF was employed as electrolyte. For LIBs, Li metal and Celgard 2400 pp membrane were used as anode and separator, respectively. 1 m LiTFSI in 1,3‐dioxolane (DOL) and dimethoxyethane (DME) (1:1 in volume) with 0.2 m LiNO_3_ was utilized as electrolyte. Galvanostatic charge/discharge measurements were performed on a multichannel battery testing system within a potential range of 0.1–1.95 V versus Mg/Mg^2+^. Cyclic voltammetry (CV) was tested by a CHI 660D electrochemical workstation between 0.1–1.95 V versus Mg/Mg^2+^. EIS was also conducted using a CHI 660D electrochemical workstation. The amplitude was 10 mV, and the applied frequency range was from 100 kHz to 0.01 Hz. All of the tests were carried out at ambient temperature. All the capacities were calculated based on the total mass of NiS and carbon, unless otherwise specified.

## Conflict of Interest

The authors declare no conflict of interest.

## Supporting information

Supporting Information

## Data Availability

The data that support the findings of this study are available in the supplementary material of this article.
