# Supplementary material for: Size‐Controllable Nickel Sulfide Nanoparticles Embedded in Carbon Nanofibers as High‐Rate Conversion Cathodes for Hybrid Mg‐Based Battery
Source: Adv Sci (Weinh). 2022 Mar 3;9(13):2106107. doi: 10.1002/advs.202106107 (PMC9069199; doi:10.1002/advs.202106107)
Supplement: Supplementary file 1 — Supporting Information [file ADVS-9-2106107-s001.pdf]

ADVANCED SCIENCE
Open Access

Supporting Information

for *Adv. Sci.*, DOI 10.1002/advs.202106107

Size-Controllable Nickel Sulfide Nanoparticles Embedded in Carbon Nanofibers as High-Rate Conversion Cathodes for Hybrid Mg-Based Battery

*Guilei Zhu, Guanglin Xia, Hongge Pan and Xuebin Yu**

Supporting Information

Size-controllable nickel sulfide nanoparticles embedded in carbon nanofibers as high-rate conversion cathodes for hybrid Mg-based battery

*Guilei Zhu, Guanglin Xia, Hongge Pan, Xuebin Yu**

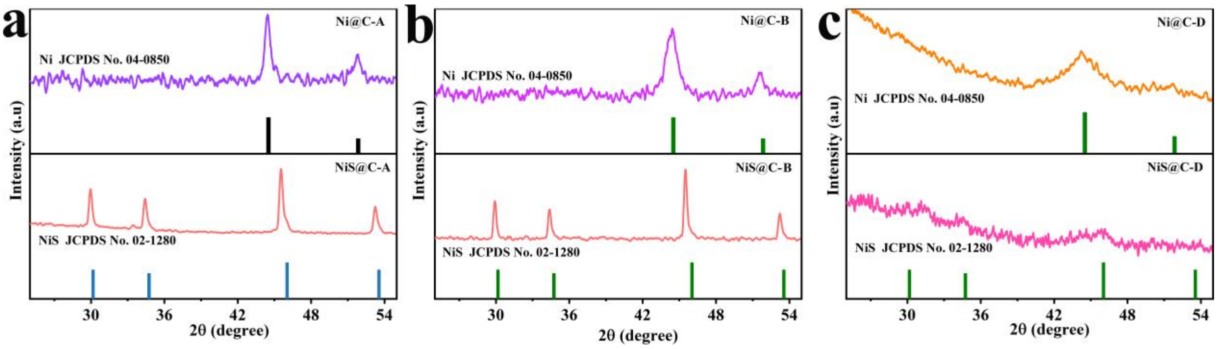

Figure S1. The XRD patterns of (a) Ni@C-A and NiS@C-A, (b) Ni@C-B and NiS@C-B, and (c) Ni@C-D and NiS@C-D.

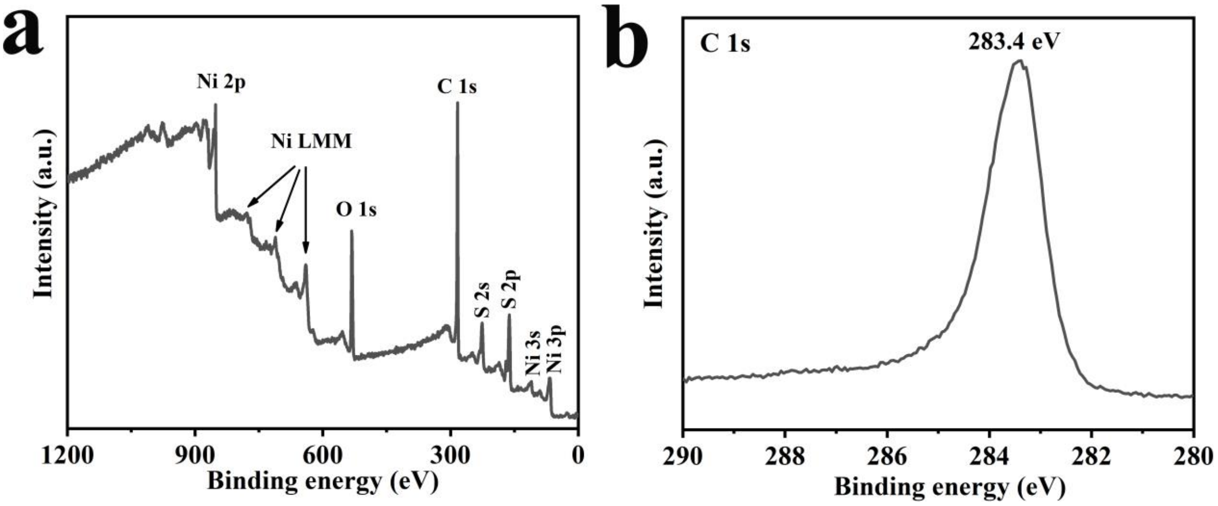

Figure S2. (a) XPS survey spectrum of NiS@C-C. (b) XPS spectrum for NiS@C-C in the C 1s region.

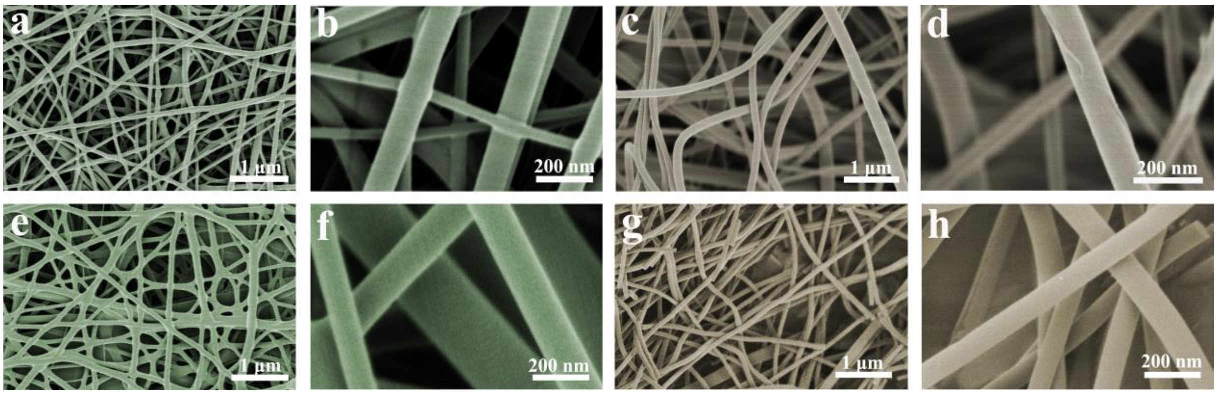

Figure S3. SEM images of (a,b,e,f) raw fibres and (c,d,g,h) oxidized fibres with different Ni source mass (g/10 ml): (a-d) 0.404 g, (e-h) 0.606 g.

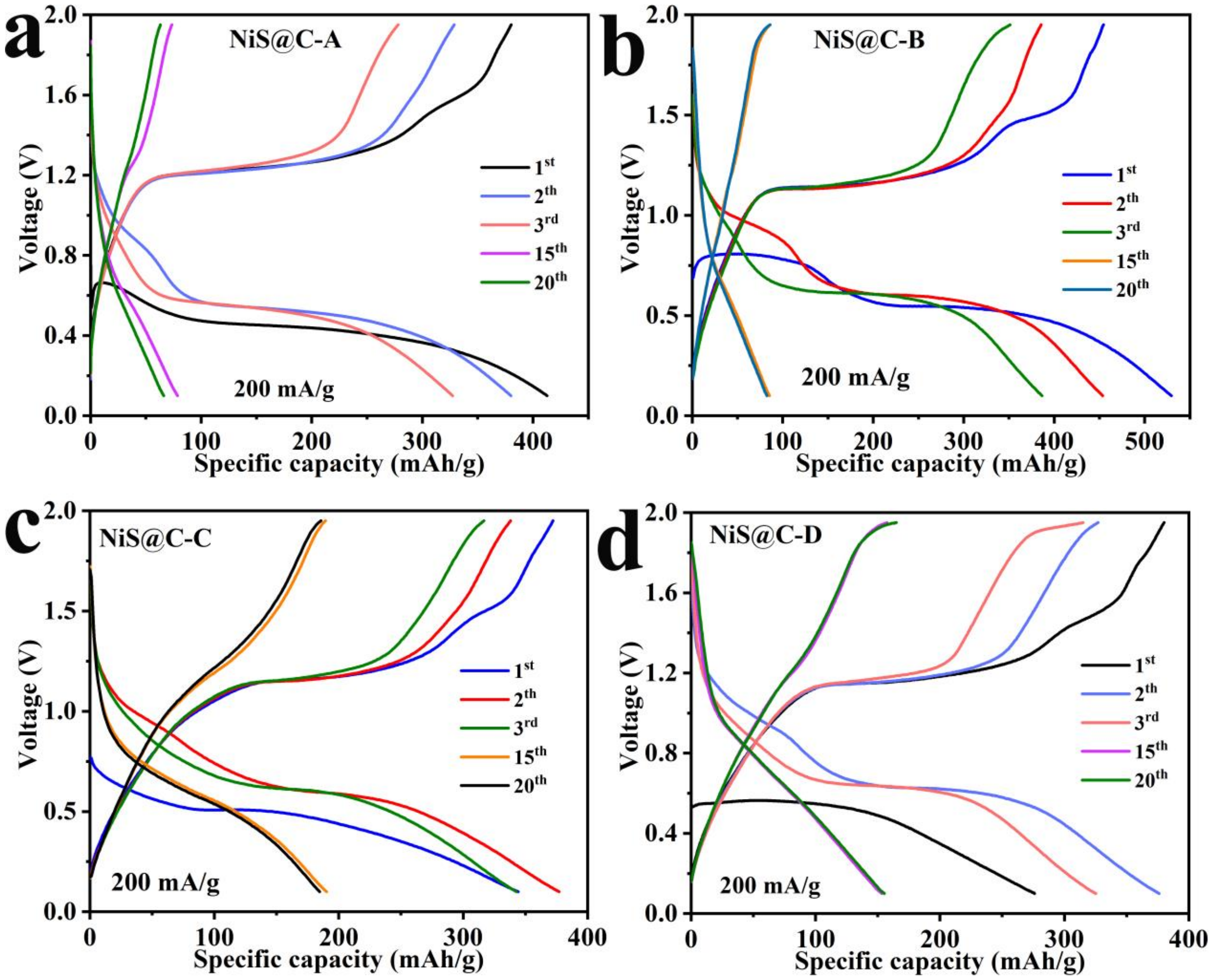

Figure S4. The galvanostatic charge−discharge voltage profiles of (a) NiS@C-A, (b) NiS@C-B, (c) NiS@C-C and (d) NiS@C-D at 200 mA g^{-1}.

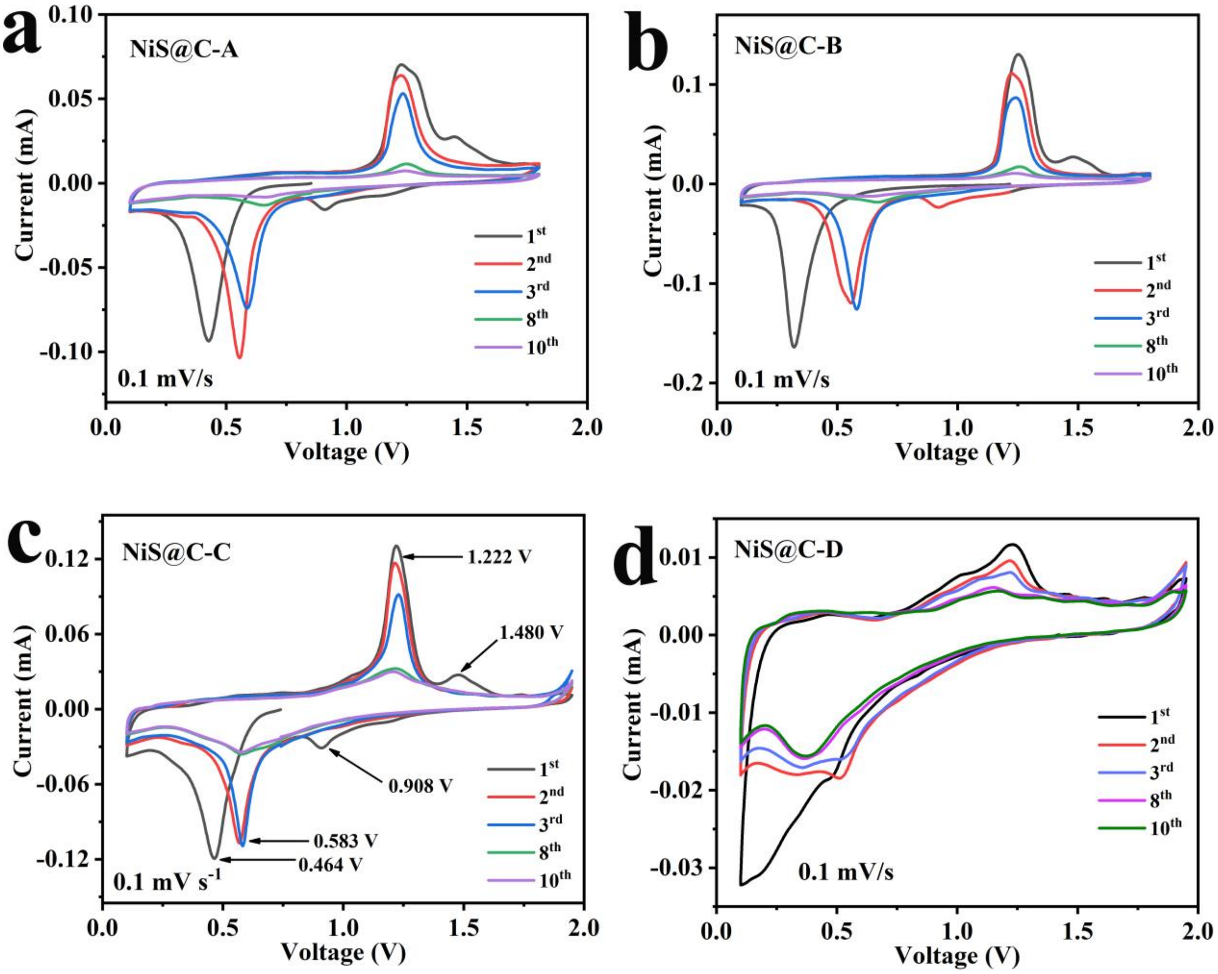

Figure S5. The CV curves of (a) NiS@C-A, (b) NiS@C-B, (c) NiS@C-C and (d) NiS@C-D at 0.1 mV s^{-1}.

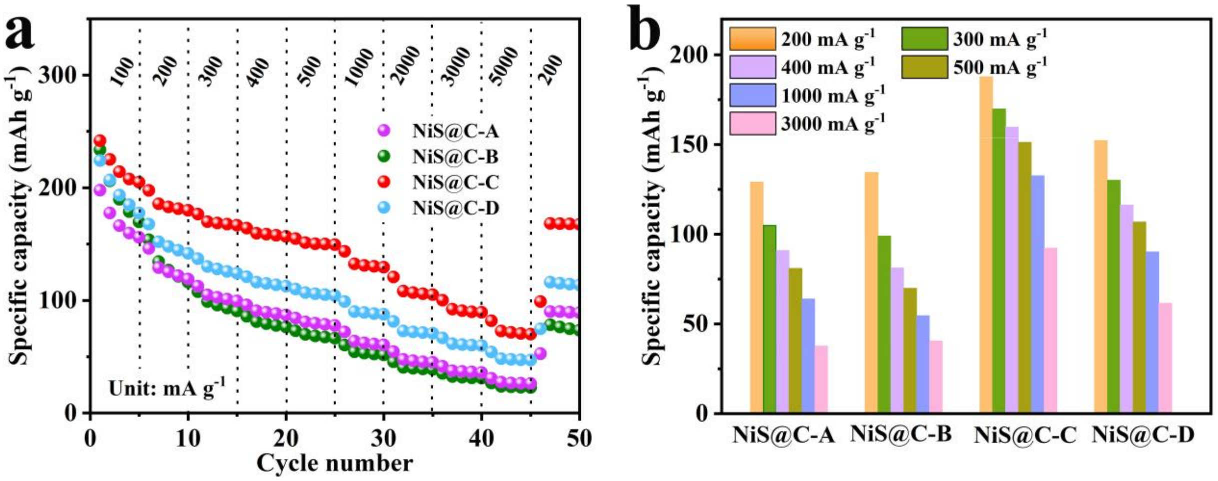

Figure S6. Rate capabilities of four cathode electrodes at various current densities.

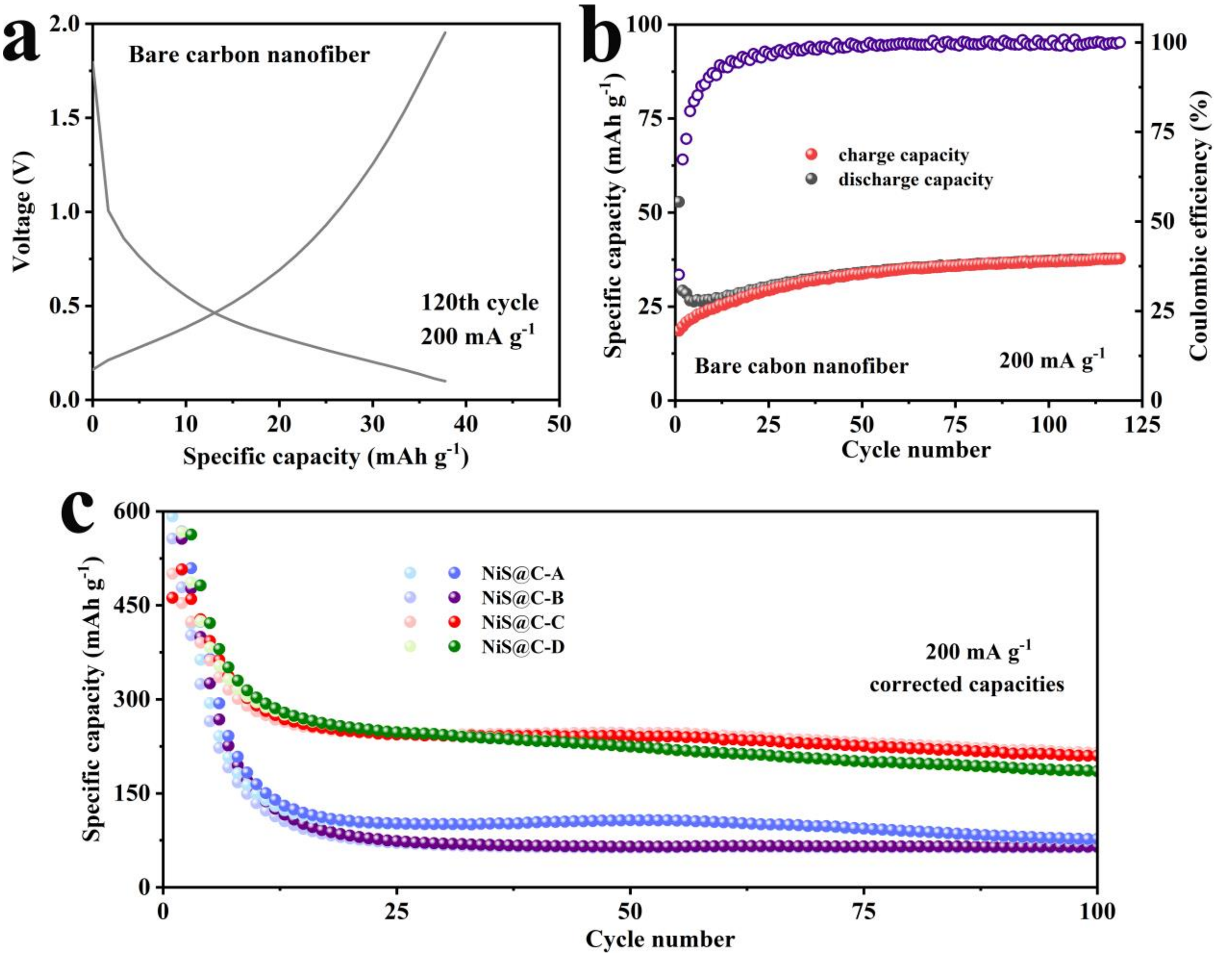

Figure S7. (a) The discharge-charge profile and (b) cycling performance of bare carbon nanofiber in MLIBs at 200 mA g^{-1}. (c) The cycling performance of NiS@C-A, NiS@C-B, NiS@C-C and NiS@C-D corrected by the property of bare carbon nanofiber and the carbon content.

The capacities of NiS@C-A, NiS@C-B, NiS@C-C and NiS@C-D were corrected via the following formula: $C_{NiS} = (C_{total} - Ccarbon*n)/(1-n)$

Where the C_{NiS}, C_{total} and Ccarbon represent corrected capacity of cathodes, the capacity based on the total mass and the capacity of bare carbon nanofiber, respectively. Besides, the n is the carbon ratio of cathodes.

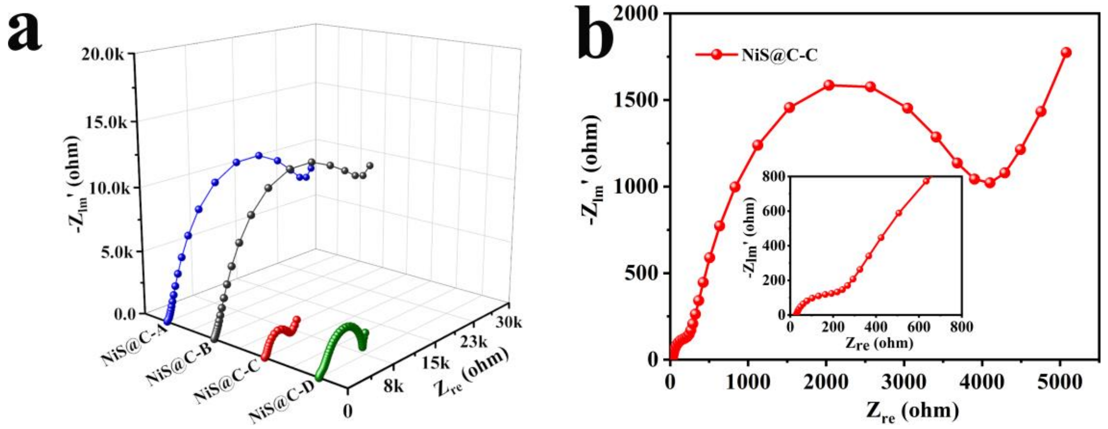

Figure S8. (a) The comparison of EIS plots for NiS@C-A, NiS@C-B, NiS@C-C and NiS@C-D. (b) The EIS plot of NiS@C-C (inset: corresponding high-frequency region).

Three factors that influence the resistance value can be summarized, including crystallinity, particle size and carbon content. The resistance values of NiS@C-A and NiS@C-B are relatively similar, which can be generated by the complementarity of NiS particle diameter and carbon content. The resistance value of NiS@C-D is slightly lower than that of NiS@C-C that may be due to the poor crystallinity resulted in lower sulfuration temperature.

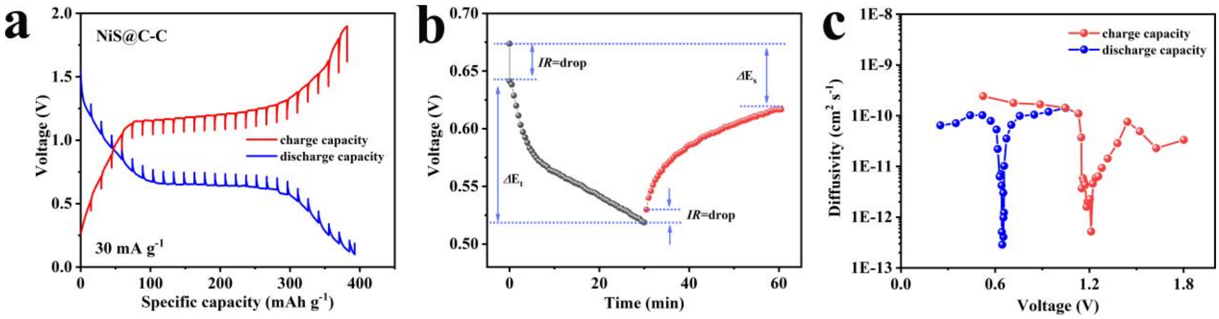

Figure S9. (a) GITT profile of NiS@C-C at a constant current pulse of 30 mA g^{-1} for 30 min followed by a rest time of 30 min. (b) The voltage profile during a single GITT step. (c) Corresponding calculated Mg^{2+}/Li^{+} diffusion coefficients.

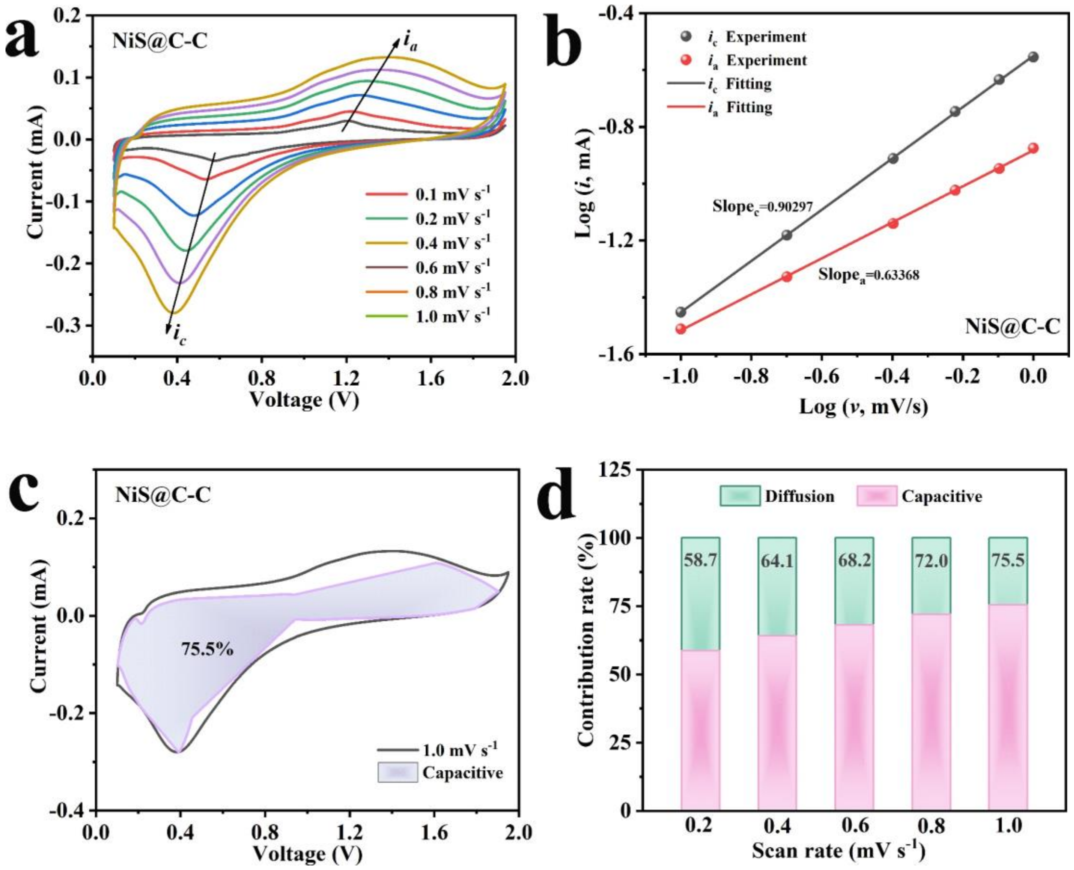

Figure S10. (a) CV curves at various scan rates from 0.1 to 1.0 mV s^{-1}. (b Corresponding log(i) versus log(v) plots of the NiS@C-C cathode. (c) Separation of capacitive and diffusion contribution at 1.0 mV s^{-1}. (d) Percentages of capacitive contribution at different scan rates.

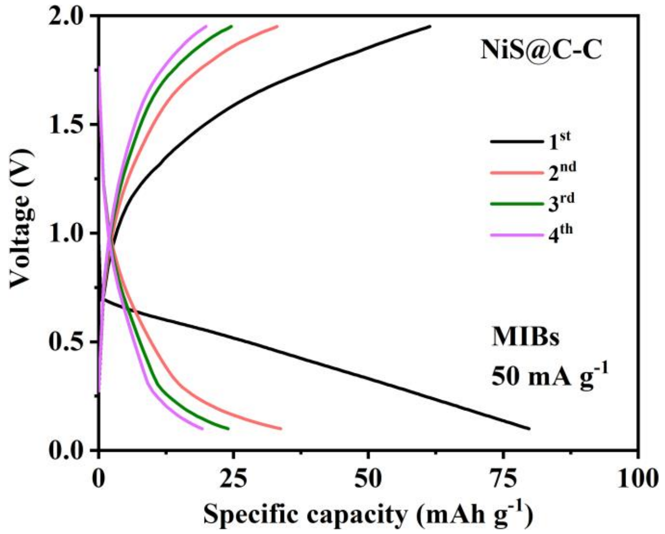

Figure S11. The charge-discharge profiles of NiS@C-C in MIBs at 50 mA g^{-1}.

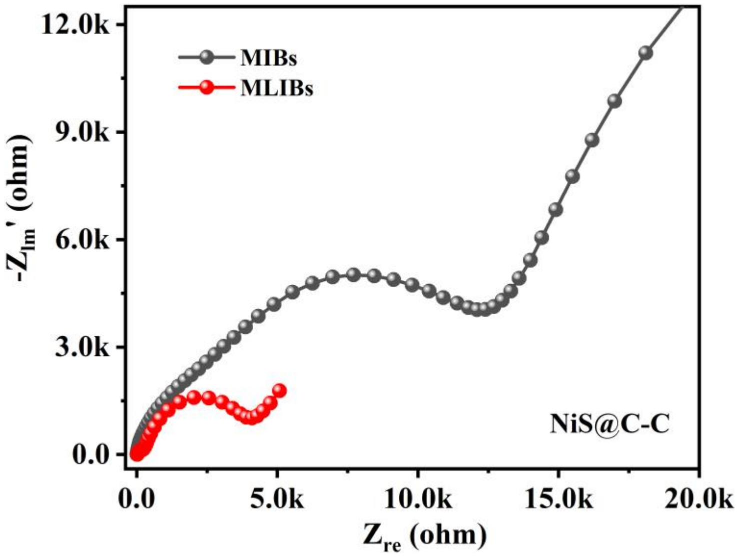

Figure S12. The comparison of EIS plots for NiS@C-C in MIBs and MLIBs

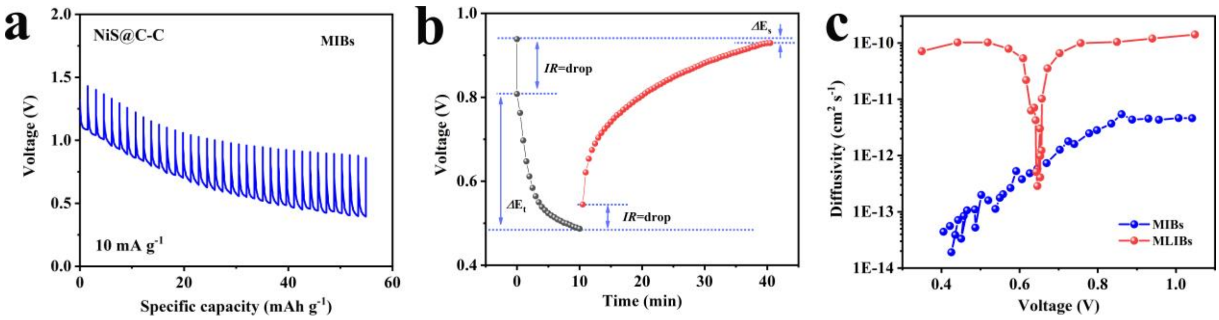

Figure S13. (a) GITT profile of NiS@C-C at a constant current pulse of 10 mA g^{-1} for 10 min followed by a rest time of 30 min. (b) The voltage profile during a single GITT step. (c) The comparison of calculated diffusion coefficients in MIBs and MLIBs in the first discharge process.

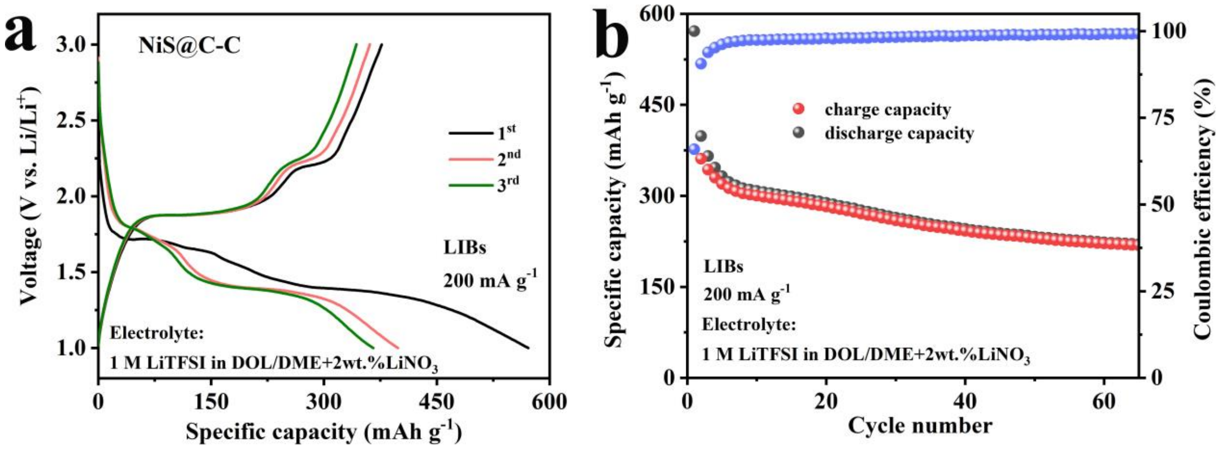

Figure S14. (a)The charge-discharge profiles and (b) cycling performance of Li||NiS@C-C at 200 mA g^{-1} in a voltage range of 1.0-3.0 V. 1 M LiTFSI in DOL/DME + 2wt.% $LiNO_3$ was employed as electrolyte.

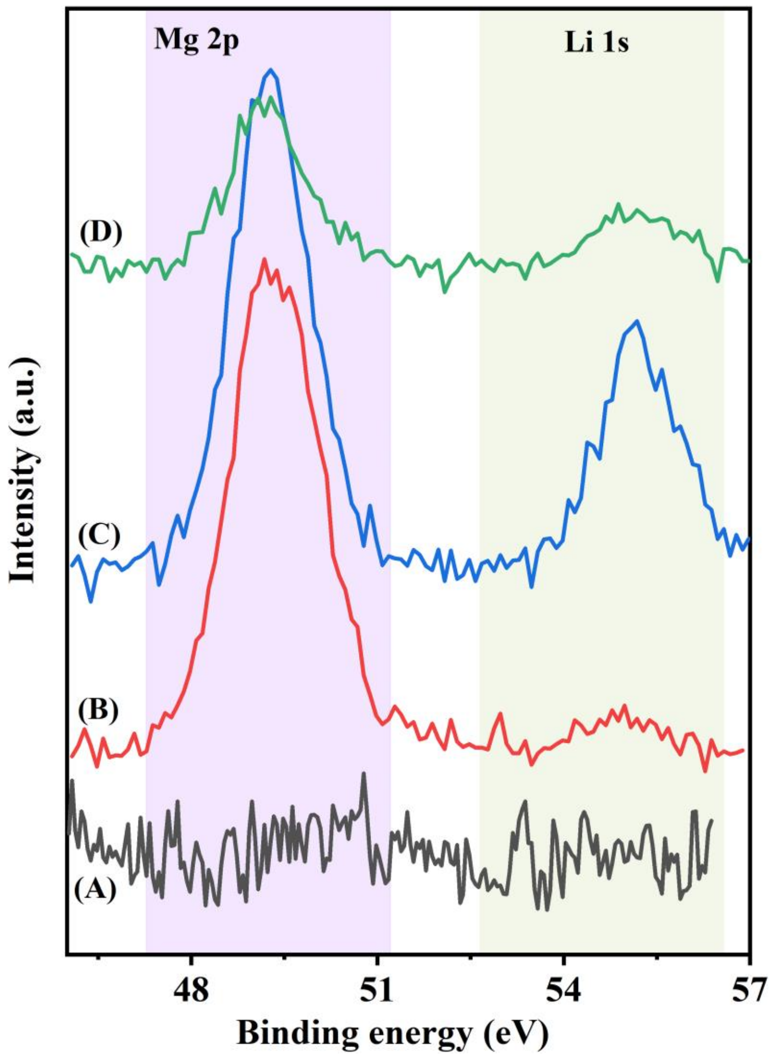

Figure S15. The high-resolution XPS spectra of Li 1s and Mg 2p at various electrochemical states: (A), as-prepared, (B), first discharge to 0.7 V, (C), first discharge to 0.1 V, (D), first charge to 1.9 V.

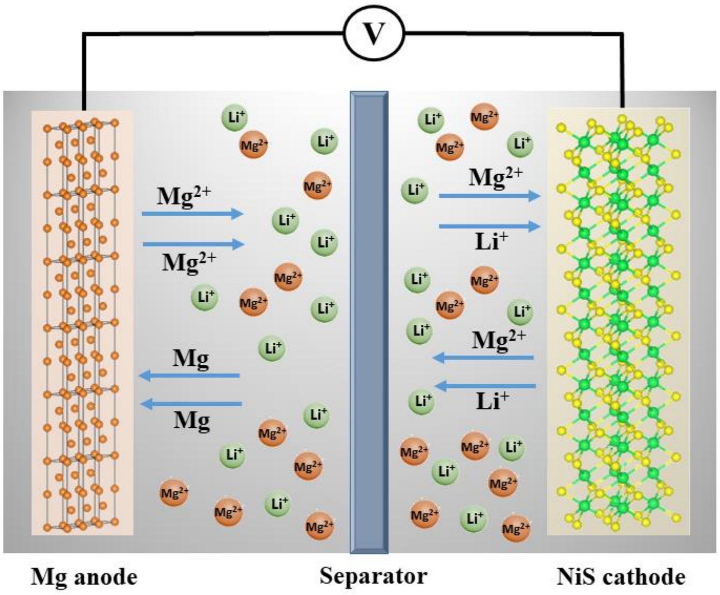

Figure S16. Schematic illustration of the hybird MLIBs designed in this work.

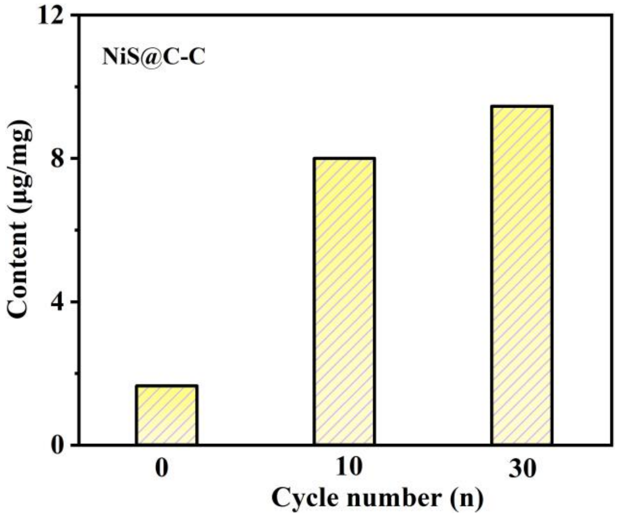

Figure S17. The mass ratios of sulfur element dissolved in electrolyte for NiS@C-C cathode material after various cycles at 200 mA g^{-1}. The cycle number of 0 is on the behalf of that the battery undergoes a rest of 24 h. The content value represents the mass of sulfur in the separator divided by the mass of S in the NiS@C-C cathode.

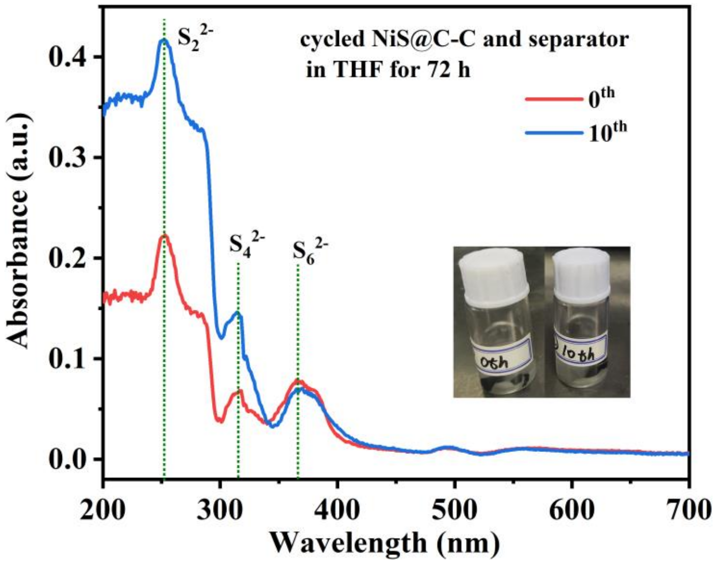

Figure S18. The UV-vis absorption spectra of the solution after soaking the cycled separator and sulfide electrodes for 72 h. The cycle number of 0 is on the behalf of that the battery undergoes a rest of 24 h.

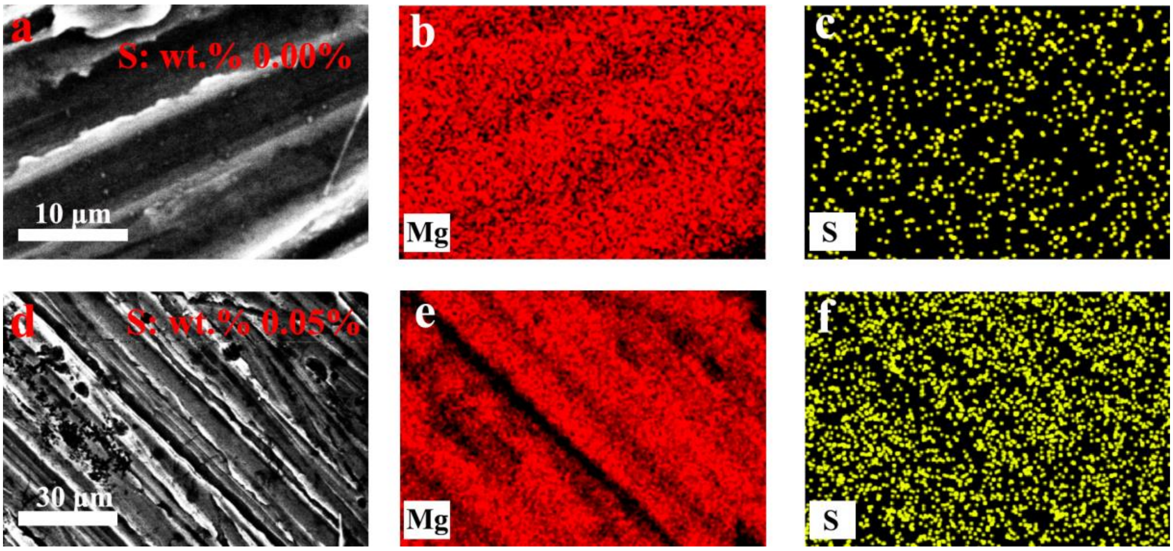

Figure S19. The SEM image of Mg anode near the separator side after cycling test for NiS@C-C: (a) 0th and (d) 10th. Corresponding elemental mappings of Mg and S: (b,c) 0th and (e,f) 10th.

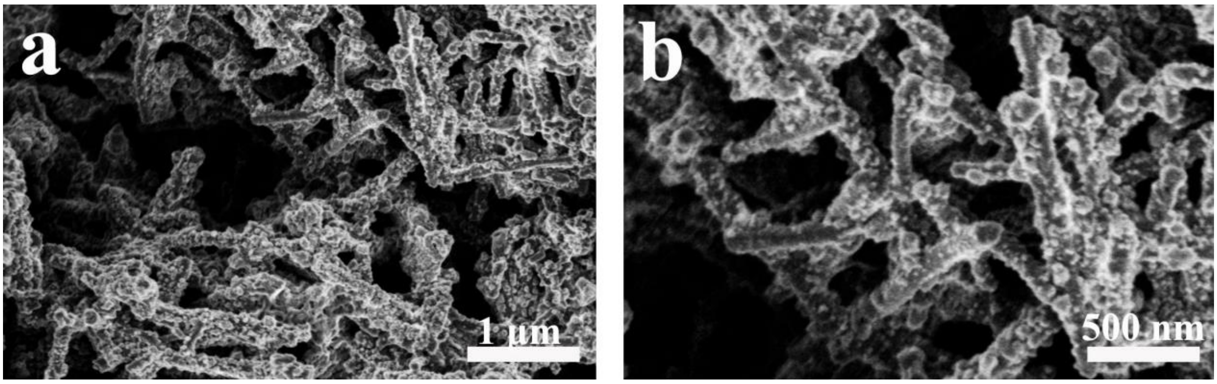

Figure S20. SEM image NiS@C-D after long time cycle.

Table S1. The detailedly synthetic conditions of NiS@C-A, NiS@C-B, NiS@C-C and NiS@C-D.

	**Ni source mass (g/10 ml)**	**Carbonization temperature (℃)**	**Sulfuration temperature (℃)**	**Sulfuration time (h)**
Bare cabon nanofiber	0 g	400	-	-
NiS@C-A	0.404 g	500	500	3
NiS@C-B	0.606 g	500	600	2
NiS@C-C	0.606 g	400	600	2
NiS@C-D	0.404 g	400	500	2
